# A rule-based kinetic model of RNA polymerase II C-terminal domain phosphorylation

**DOI:** 10.1098/rsif.2013.0438

**Published:** 2013-09-06

**Authors:** Stuart Aitken, Ross D. Alexander, Jean D. Beggs

**Affiliations:** 1 MRC Human Genetics Unit, IGMM, University of Edinburgh, Edinburgh EH4 2XU, UK; 2 Wellcome Trust Centre for Cell Biology, University of Edinburgh, Edinburgh EH9 3JR, UK; 3 SynthSys, University of Edinburgh, Edinburgh EH9 3JD, UK

**Keywords:** rule-based modelling, Kappa, transcription

## Abstract

The complexity of many RNA processing pathways is such that a conventional systems modelling approach is inadequate to represent all the molecular species involved. We demonstrate that rule-based modelling permits a detailed model of a complex RNA signalling pathway to be defined. Phosphorylation of the RNA polymerase II (RNAPII) C-terminal domain (CTD; a flexible tail-like extension of the largest subunit) couples pre-messenger RNA capping, splicing and 3′ end maturation to transcriptional elongation and termination, and plays a central role in integrating these processes. The phosphorylation states of the serine residues of many heptapeptide repeats of the CTD alter along the coding region of genes as a function of distance from the promoter. From a mechanistic perspective, both the changes in phosphorylation and the location at which they take place on the genes are a function of the time spent by RNAPII in elongation as this interval provides the opportunity for the kinases and phosphatases to interact with the CTD. On this basis, we synthesize the available data to create a kinetic model of the action of the known kinases and phosphatases to resolve the phosphorylation pathways and their kinetics.

## 1. Introduction

Ordinary differential equation and stochastic modelling techniques are applicable to RNA processing and signalling pathways. However, a significant obstacle must be overcome: the explosion of entities and reactions that follows when RNA species, their precursors, complexes and states are represented. Rule-based modelling has previously been shown to address combinatorial complexity in protein interactions [1,2]; here, we explore the potential of this new methodology for modelling RNA metabolic pathways. Rule-based models in the Kappa language that we adopt [3] are simulated stochastically, and thus the approach accounts for low molecule numbers and the discrete states that molecules can adopt. We discuss transcription by RNA polymerase II (RNAPII) as an example of a complex system that can be modelled in detail using a rule-based approach.

We present a novel rule-based model of transcription that includes the phosphorylation states of multiple heptapeptide repeats in the representation of the RNAPII enzyme. The model represents the kinases and phosphatases that act on these repeats during initiation, elongation, 3′ end maturation and RNAPII recycling. We demonstrate that this model can help explain both genome-wide patterns of RNAPII phosphorylation on yeast genes, and changes in phosphorylation that take place when an intron-containing yeast reporter gene is induced.

All protein-coding genes and most small non-coding RNAs are transcribed by RNAPII [4]. The C-terminal domain (CTD) of the largest subunit of RNAPII contains the heptapeptide repeat, Y_1_S_2_P_3_T_4_S_5_P_6_S_7_, and functions in coupling transcription to mRNA processing and chromatin modifications [5,6]. In yeast, the CTD consists of 26 repeats of this heptad (52 in humans), each of which can be phosphorylated at the serines (S) in positions 2, 5 and 7, as well as at the tyrosine (Y; position 1) and threonine (T; position 4). In addition, the proline (P) at position 6 (at least) undergoes isomerization between *cis*- and *trans*-configurations, yielding a substrate with a vast potential for signalling [7]. Synthetic CTD peptides have been shown to bind proteins with a wide range of functions, including Set2, Ssd1, Prp40, Hrr25 and Ess1, with a specificity that varies significantly with the pattern of serines 2 and 5 phosphorylation on the heptad [8]. CTD phosphorylation facilitates recruitment of capping factors at the 5′ end of genes, 3′ end processing factors and certain splicing factors *in vivo* [7,9].

In this paper, we synthesize the *in vivo*, *in vitro* and biochemical evidence on the action, substrate and context specificity of the known CTD kinases and phosphatases to construct a kinetic model of the RNAPII CTD cycle, focusing on the phosphorylation of serines 2, 5 and 7. Formalizing the reactions and kinetics of this key RNA signalling pathway has the benefit of making all assumptions explicit and easy to scrutinize. The Kappa rule-based approach to modelling that we adopt [3] makes the assumptions particularly easy to communicate, and simplifies the process of extending the model to include additional factors and modifications as they are discovered.

Chromatin immunoprecipitation (ChIP) analysis is the prevalent technique for mapping the extent of RNAPII phosphorylation with respect to genes *in vivo*. Modelling can assist the interpretation of ChIP data by accounting for the substrate specificity of the antibodies used [10], and by distinguishing the changes in phosphorylation due to signalling from those due to RNAPII accumulation should the polymerase pause on the DNA. The development of the formal model was motivated by the desire to interpret genome-wide ChIP data [11], and kinetic data linking CTD signalling to a splicing-related pause observed in yeast [12]. Data from each of these experiments are presented to illustrate the use of the model.

## 2. The C-terminal domain kinases and phosphatases

**Figure 1.**
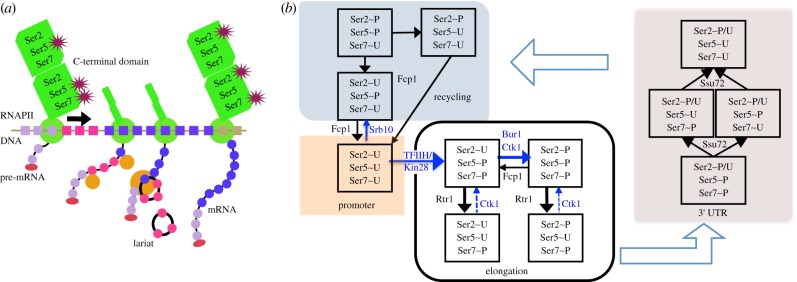
Phosphorylation of serines 2, 5 and 7 (Ser2, Ser5 and Ser7) on the CTD of RNAPII. (*a*) Ser5 and Ser7 are phosphorylated at the promoter by TFIIH/Kin28. Ser2 phosphorylation increases towards the 3′ end of the gene and is a requirement for 3′ end maturation. Serine phosphorylation has been linked to cotranscriptional splicing (the excision of an intron, in red, is illustrated). (*b*) The actions of the kinases Kin28, Ctk1, Bur1 and Srb10, and the phosphatases Rtr1, Fcp1 and Ssu72 on an individual heptapeptide are indicated by a change of state of one of Ser2, Ser5, Ser7 to P from U, or vice versa. The specific actions of these proteins at the promoter, during elongation, at the 3′ UTR and on the free RNAPII (recycling) must be distinguished. Higher reaction rates are indicated by broader arrows. Two reactions supported by biochemical evidence but not by *in vivo* data are indicated by dashed arrows.

The process of messenger RNA transcription, the phases of the CTD cycle and the various states that an individual heptapeptide repeat can adopt in each phase are illustrated in figure 1. In addition to summarizing the rule-based model, figure 1*b* serves as a reference for the following survey of proteins, their activity as kinases or phosphatases and their substrate specificities. The actions of these entities (referred to as agents in Kappa terminology) are represented as transitions (arrows) between states of the heptapeptide.

Yeast kinase Kin28 phosphorylates RNAPII CTD on serine 5 (Ser5) after the assembly of the preinitiation complex (PIC) at the promoter [6] (denoted by the arrow labelled TFIIH/Kin28 in figure 1*b*). The phosphorylation of the CTD on Ser5 is important for the recruitment of the mRNA capping enzyme complex, which may contribute to the processivity of the elongation complex. Kin28 and Srb10 are indistinguishable in terms of their ability to phosphorylate the CTD on Ser5, yet Kin28 is a positive regulator, whereas Srb10 is a negative regulator of transcription. The phosphorylation of the CTD by Srb10 prior to the formation of a stable PIC is proposed to inhibit transcription, thus it is the timing of CTD phosphorylation that determines the mode of regulation [13]. In figure 1*b*, Srb10 competes with Kin28 to prevent RNAPII activity as both kinases act on completely dephosphorylated substrates (Ser2∼U, Ser5∼U, Ser7∼U) at the promoter.

Both serine 2 (Ser2) kinase Ctk1 and phosphatase Fcp1 can be cross-linked to promoter and coding regions, but not to the non-transcribed regions, and they have opposing effects on elongating polymerases [14]. Fcp1 also has a role in the recycling (dephosphorylation) of RNAPII for subsequent rounds of transcription.

Biochemical investigation of the specificity of Ctk1 shows this kinase to react with synthetic repeats phosphorylated at either Ser5 or Ser2 to phosphorylate Ser2 and Ser5, respectively. Unphosphorylated repeats are phosphorylated at Ser5 by Ctk1, but to a lesser extent [10].

Biochemical analysis of Fcp1 from *Schizosaccharomyces pombe* reveals this phosphatase to have greater activity on synthetic heptad repeats phosphorylated on Ser2 than on Ser5 [15], and phosphate is efficiently released by Fcp1 from doubly phosphorylated repeats [15,16]. Fcp1 directly recognizes CTD repeats, in addition to binding to RNAPII at a functionally distinct site that may mediate its effect on elongation [16]. The dephosphorylation of Ser5 by Fcp1 after the termination of transcription, *in vitro*, can be explained by the lower specificity of Fcp1 for a ternary complex of DNA, RNAPII, TFIIH in comparison with that for free RNAPII [17]. Consequently, Fcp1 is modelled as acting on Ser2∼P during elongation and on both Ser2∼P and Ser5∼P during recycling.

The Ser2 kinase Bur1 is recruited to CTD repeats of elongating RNAPII phosphorylated on Ser5. Bur1 augments the major Ser2 kinase (Ctk1) and is capable of phosphorylating CTD repeats at Ser2, when Ctk1 is inactivated [18]. Thus, during elongation, Bur1 acts only on repeats phosphorylated on Ser5, whereas Ctk1 potentially acts on three of the four possible CTD states (figure 1*b*).

Rtr1 is a Ser5 phosphatase that localizes to DNA coding regions [19]. Rtr1 regulates the transition in the phosphorylation of the CTD that takes place between the promoter, where serines 5 and 7 are phosphorylated, and around 1000 nt from the transcription start site (TSS), by which point Ser2 phosphorylation has increased and Ser5 phosphorylation has diminished [19]. Rtr1 mutants show a large increase in Ser5 phosphorylation, indicating a loss of phosphatase activity, and an increase in Ser2 phosphorylation in the coding region relative to the wild-type. If Ser2 phosphorylation primarily occurs through repeats that are unphosphorylated at both serines 2 and 5 (i.e. through the state that results from Rtr1 phosphatase action downstream of the promoter) then Ser2 phosphorylation would be expected to reduce considerably, rather than increase in a Rtr1 mutant. This finding, together with the lack of biochemical evidence to support the generation of Ser2 phosphorylation from doubly unphosphorylated repeats, indicates that Rtr1 may compete kinetically with the Ctk1 and Bur1 CTD kinases to regulate the Ser5 to Ser2 transition by removing their preferred substrate.

A recent report has failed to identify an active site on Rtr1 [20]. Experiments to demonstrate CTD phosphatase activity were unsuccessful which suggests that Rtr1 may have a non-catalytic role in CTD dephosphorylation [20]. Thus we consider a complex involving Rtr1 to mediate the Ser5∼P to Ser5-U reaction, and refer to this complex as Rtr1 for simplicity. In summary, during elongation Rtr1 acts on both CTD states where Ser5 is phosphorylated (figure 1*b*).

Ssu72 is a component of the cleavage/polyadenylation factor complex that localizes predominantly to the 3′ end of genes [21], and a CTD phosphatase capable of dephosphorylating Ser5 on singly and doubly phosphorylated repeats *in vitro* [22]. Ssu72 may dephosphorylate the CTD at the 3′ end of genes to recycle the RNAPII into an initiation-competent form [22]. It is also proposed to be a transcription elongation factor, mediated by its activity as a CTD phosphatase and its physical interaction with TFIIB, TFIID, TFIIH and RNAPII [23].

A recent study by Bataille *et al.* [11] confirms Kin28 to be a serine 7 (Ser7) kinase [24] and Ssu72 to be a Ser7 phosphatase. Antibodies specific for Ser2 (H5 and 3E10), for Ser5 (3E8 and H14) or for Ser7 (4E12) were used to compose ‘metagene’ profiles of CTD phosphorylation across the yeast genome. The patterns of Ser5 dephosphorylation and Ser2 phosphorylation are the same in long and short genes: the changes in phosphorylation are a function of distance from the transcription start site (or equivalently, of time spent in elongation) rather than as a function of the proportion of the gene transcribed. The level of Ser2 phosphorylation is less at the end of short genes than at the end of long genes owing to the reduced opportunity for kinases to act. Serines 5 and 7 are phosphorylated with the same kinetics near the promoter. Serine 5 phosphorylation reduces over the subsequent 750 nt, while Ser2 phosphorylation accumulates with slower kinetics to reach a plateau around 1000 nt from the TSS [11]. The data suggest that the polyA site is the trigger for the dephosphorylation of serines 5 and 7 at the 3′ end [11].

## 3. Methods

A model of the events in RNAPII elongation necessarily includes the positions that RNAPII may occupy on the DNA (for example, to match target ChIP sites), and must account for the possible patterns of phosphorylation that each heptapeptide repeat can adopt. If *N* positions of RNAPII on the DNA are included in the model, and assuming only two repeats of serines 2 and 5 are represented (giving 2^2^ × 2^2^ = 16 combinations of phosphorylation states), the number of species of RNAPII to be modelled is 16 × *N*, and the number of possible transitions is 16 × 4 × (*N* – 1) (assuming each of the 16 states can change in four possible ways). In a conventional model, rather than representing a single species with multiple states, we must represent multiple species, resulting in unacceptable model complexity.

Rule-based modelling in Kappa [3,25,26] provides a solution to this problem as rules, representing binding/unbinding reactions, need only match a partial description of the state of an agent, and only modify a subset of the bonds between agents.

Entities are referred to as agents in Kappa, and written RNAPII() DNA(). Agents have named sites through which they interact; for example, p can represent the promoter in DNA: DNA(p); and agents form complexes by creating bonds between sites. To state that RNAPII is bound to DNA at the promoter we can write RNAPII(a!1),DNA(p!1), where the bond 1 is shared by sites a and p (expressed as a!1 and p!1 within the respective agents).

Sites can have states, for example, RNAPII phosphorylated at Ser5 but not Ser2 would be written RNAPII(Ser2∼U,Ser5∼P), and rules can depend on the phosphorylation of RNAPII at one site, and can include or ignore the state of other sites and the position of the RNAPII on the DNA as required. The binding of Rtr1 to RNAPII at the Ser5 site, and the subsequent dephosphorylation of this site and release of Rtr1 can be expressed by two rules of the form precondition -> postcondition @ rate as follows:

Rtr1(a), RNAPII(a!_,e,Ser5∼P) ->

Rtr1(a!1), RNAPII(a!_,e,Ser5∼P!1) @0.1

Rtr1(a!1), RNAPII(Ser5∼P!1) ->

Rtr1(a), RNAPII(Ser5∼U) @1

The first rule specifies the substrate specificity (Rtr1 binds phosphorylated Ser5) and limits the applicability of the rule to the coding region where RNAPII is bound to some site on the DNA() (by the inclusion of RNAPII(a!_)) excluding the 3′ UTR (by the inclusion of RNAPII(e) as e is always bound when RNAPII is at the 3′ UTR). The substrate specificity of the other kinases and phosphatases may place conditions on Ser2, Ser5 and Ser7 as necessary. (The complete set of 56 rules for phosphorylation is provided in the electronic supplementary material, file phosphorylation.ka.)

The proposed model represents 80 positions on the DNA that can be occupied by RNAPII. These are encoded as 80 binding sites on the DNA agent. Each site has an associated rule that specifies when RNAPII moves to the subsequent site. The phosphorylation rules apply to RNAPII independently of the site it occupies on the DNA, hence combinatorial explosion is avoided. In contrast, a species-based model would require 1280 species and 5056 reactions to encode two repeats each capable of adopting four states at 80 locations.

Rules in the Kappa language also allow new entities to be created. This is particularly useful in modelling RNA pathways as transcription adds nucleotides to the nascent RNA as the polymerase traverses the gene. The synthesis of splice sites, the branchsite, splicing enhancer and splicing suppressor sites all provide the substrates for reactions that may be concurrent with transcription. These reactions determine the final sequence of the RNA and the protein product. Kappa provides a readily understandable rule language, and tools for stochastic model simulation, facilitating model development. Alternative rule-based languages that provide comparable support include BioNetGen, NFSim and ML-Rules [27–29]. The promise of these approaches is described further in Bachman & Sorger [2].

## 4. Results

A detailed Kappa model of 219 rules describing the entire transcription pathway has been constructed (see figure 1, §6 and electronic supplementary material, file model.tar.gz). Defining the transcription pathway allows rules specifying the interaction of the kinases and phosphatases with sites on the CTD of RNAPII to take the context of the CTD into account as it progresses through the transcription cycle. As discussed earlier, these proteins may act on the CTD in specific ways at the promoter, during elongation, at the 3′ UTR and on free RNAPII, and hence the model must distinguish these contexts. The states that an individual heptad may adopt, and the transitions between them, are indicated in figure 1*b* for each of the four contexts.

We now consider the phosphorylation patterns in two different kinds of yeast data: metagene profiles obtained from genome-wide data [11], and kinetic data obtained from the induction of a reporter gene [12]. As the reporter gene contains a single intron (for the study of splicing), we focus on single-intron genes in the genome-wide analysis.

### 4.1. Metagene profiles

**Figure 2.**
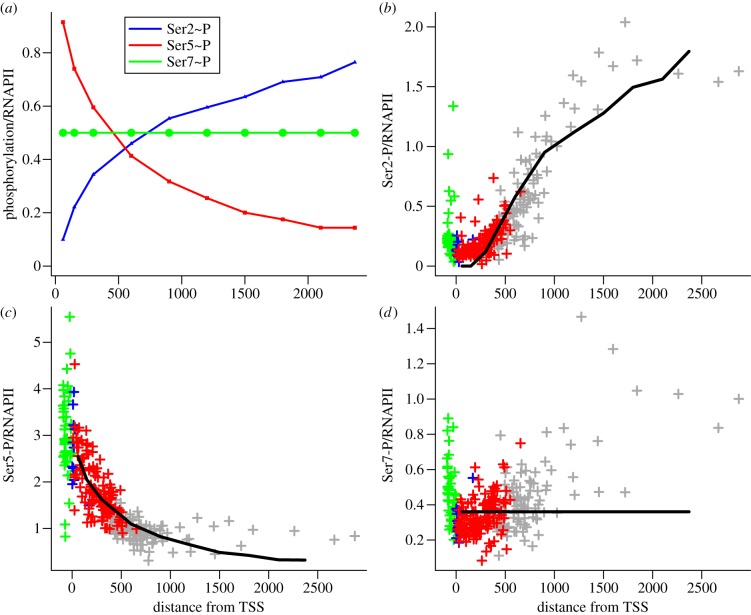
Ser2, Ser5 and Ser7 phosphorylation in the coding regions of single-intron genes. (*a*) Model predictions of the ratios of Ser2∼P, Ser5∼P and Ser7∼P to RNAPII. (*b–d*) Scatter plots of the ratios of Ser2∼P, Ser5∼P and Ser7∼P to RNAPII genome-wide for genes with introns between 300 and 600 nucleotides in length derived from the data in [11]. (See electronic supplementary material, figure S1 for intron and exon length distributions.) Data sampled from the promoter region (100 nucleotides upstream from the transcription start site (TSS)) are plotted in green, exon 1 (blue), the intron (red) and exon 2 (grey). In (*b–d*), the black lines indicate the model simulations of (*a*) thresholded and scaled to best approximate the measured phosphorylation.

Genome-wide profiling of highly expressed genes shows Ser5∼P to reduce rapidly towards a steady state as RNAPII leaves the promoter, and Ser2∼P to increase [11]. We find that this pattern also occurs in yeast genes with a single intron where the intron is long (300–600 nucleotides; figure 2). This subset of the genome is highly expressed on average. The distributions of exon, intron and gene lengths in single-intron yeast genes are plotted in the electronic supplementary material, figure S1, and the CTD phosphorylations of all highly expressed genes are presented as a scatter plot in the electronic supplementary material, figure S2, for comparison with figure 2*b–d* (data from [11]).

The Kappa model of the CTD cycle is capable of reproducing the observed pattern of Ser2, Ser5 and Ser7 phosphorylation under different assumptions about the ability of Ctk1 to act as a Ser5 kinase during elongation (a controversial issue in RNAPII biology). These reactions have been observed *in vitro*, but *in vivo* evidence is lacking. To explore the inclusion, or otherwise, of the possible Ctk1 Ser5 kinase activity in the model, we examine the fit of four versions of the model to the genome-wide data. We consider models that lack any Ctk1 Ser5 kinase activity (see the electronic supplementary material, figure S3), include Ctk1 Ser5 kinase activity from the Ser2∼U, Ser5∼U, Ser7∼P substrate (see the electronic supplementary material, figure S4), include Ctk1 Ser5 kinase activity from the Ser2∼P, Ser5∼U, Ser7∼P substrate (see the electronic supplementary material, figure S5), and finally include Ctk1 Ser5 kinase activity from both Ser2∼U, Ser5∼U, Ser7∼P and Ser2∼P, Ser5∼U, Ser7∼P substrates (see the electronic supplementary material, figure S6). To widen the basis of model comparison, we also add three assumptions: that Rtr1 binds faster than, at the same rate or slower than the rate at which Ctk1 and Bur1 bind when acting as Ser2 kinases. For Rtr1 to mediate the Ser5∼P to Ser2∼P transition as proposed by Mosley [19], it is to be expected that the Rtr1 binding rate would be faster than that of the competing Ser2 kinases, and hence that the fit of the model to the data should be better for faster Rtr1 binding.

Qualitatively, the loss of Ser5∼P and gain of Ser2∼P along the gene is maintained under the alternative modelling assumptions. However, the kinetics of these changes differ considerably when the model predictions are mapped to the genome-wide data. The possible Ser5∼P kinase reaction from substrate Ser2∼P, Ser5∼U, Ser7∼P to Ser2∼P, Ser5∼P, Ser7∼P does not improve the fit of the model to the data, either by itself or in conjunction with that from Ser2∼U, Ser5∼U, Ser7∼P, and is not included in the model. In contrast, the addition of the reaction from Ser2∼U, Ser5∼U, Ser7∼P to Ser2∼U, Ser5∼P, Ser7∼P quantitatively improves the fit of the model to the data and maximizes the dynamic range of predicted values that correspond to observed values.^1^ Consequently, this Ctk1 Ser5 kinase reaction is included in the model henceforth.

The predictions of the model (figure 2*a*) are indicated by the solid black lines in the scatterplots of the genome-wide data in figure 2*b–d*. Ser7∼P is predicted to be constant as no Ser7 kinase or phosphatase has been proposed to act during elongation, and hence there are no rules for the modification of this residue. Figure 2*d* shows that a number of intron-containing genes have elevated Ser7∼P at the TSS and on exon2, which may indicate a regulatory role [30]. A general tendancy for Ser7∼P to increase along the gene can be seen in the electronic supplementary material, figure S2 (potentially attributable to Bur1 [31]).

In the Kappa model, changes in phosphorylation are not dependent on the length of the gene, but on the time RNAPII spends in elongation which provides the opportunity for the kinases and phosphatases to act. Hence the model provides mechanisms to explain genome-wide observations, including the proposed regulatory role of Rtr1 [19].

### 4.2. Sensitivity analysis

The model specifies that the rate at which Bur1 and Ctk1 bind to sites on RNAPII is half that of Rtr1 binding, and consequently Rtr1 tends to bind first and reduce the extent of Ser5∼P. Nonetheless, some RNAPII heptads are directly phosphorylated on Ser2 by Bur1 and Ctk1, and Ser5∼P can be restored by Ctk1 allowing these kinases to act. When these binding rates are reduced, few heptads are phosphorylated on Ser2 and, as this is a requirement for 3′ end maturation, RNAPII accumulates at the 3′ end. In contrast, when the binding rates are increased, Ser2∼P and Ser5∼P reach their steady-state values within 150 nt of the promoter (that is, more rapidly than observed).

**Figure 3.**
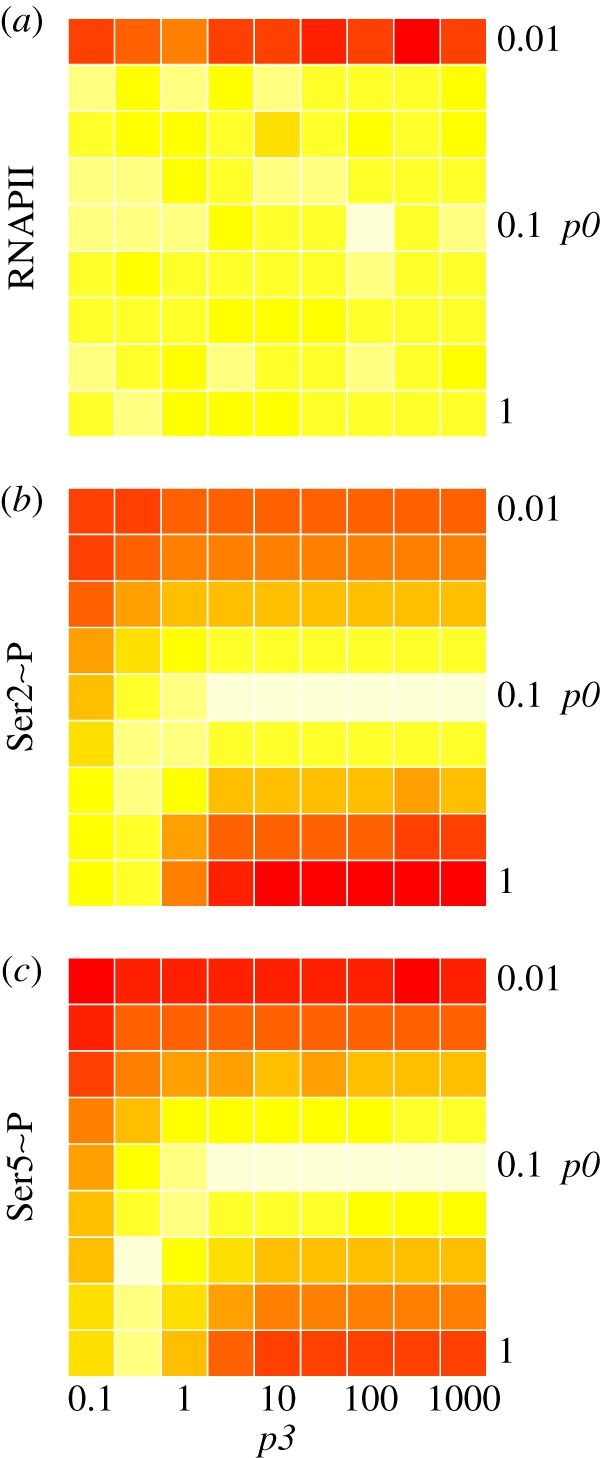
Sensitivity of (*a*) RNAPII, (*b*) Ser2∼P and (*c*) Ser5∼P to the binding rate *p0* and unbinding rate *p3*. The colour scale white to red indicates the effect on the model prediction (lowest to highest sum of square differences) for each combination of *p0* and *p3* surveyed, taking *p0* = 0.1 and *p3* = 100 as the reference.

The effects of varying the two parameters that govern the binding and release rates of the kinases and phosphatases that act during elongation were explored systematically by sensitivity analysis. Figure 3 presents heatmaps showing the difference of simulated RNAPII, Ser2∼P and Ser5∼P with respect to simulations based on the parameters used in the baseline model (figure 2). The binding rate *p0* (see §6) was varied over two orders of magnitude (0.01–1) and the unbinding rate *p3* over four orders of magnitude (0.1–1000). The binding rates for all kinases were kept at a constant fraction of *p0* as defined in the model; for example, the rate for Bur1 was kept at *p0*/2, thus *p0* acts as a ‘meta-parameter’. Similarly, all unbinding reactions occur at *p3*. All other rates were kept constant, most importantly, those governing the progression of RNAPII along the gene as this defines the window of opportunity for CTD modifications. This computational experiment determines the robustness of the predictions to parameter values.

The heatmap in figure 3*a* shows that the predicted profile of RNAPII is insensitive to the phosphorylation parameters except with values for *p0* of 0.01 or less: should there be no Ser2 phosphorylation at the 3′ end of the gene as the result of the low binding rate, the model requires RNAPII to pause as 3′ end maturation cannot proceed without Ser2∼P and this results in an accumulation of RNAPII at the 3′ end. The heatmaps for Ser2∼P and Ser5∼P show a greater sensitivity for these predictions on values of *p0* greater and less than those of the baseline model. The value of *p3* does not have an impact on the predictions while *p3* is 10 times the value of *p0* or greater. Lower values of *p3* combined with greater values of *p0* give rise to predictions comparable with those of the baseline model as the duration of the binding/unbinding sequence is comparable. Thus, the potential trade-off between binding rates illustrates the importance of direct measurement of binding constants (e.g. as in [32]) as their values cannot be inferred with confidence from model fitting.

### 4.3. Kinetic profiles

Having established that the typical profiles of intronless and intron-containing genes are similar at steady state, and that the Kappa model reproduces this profile, we apply the model to data from the induction of a single gene. Data from individual genes often provide insights into regulation and opportunity for more detailed investigations than genome-wide datasets, which typically reflect steady-state conditions.

The ChIP data on a splicing-related pause in the Ribo1 yeast gene provide an opportunity to explore the relationship between RNAPII density and serine phosphorylation [12]. Briefly, Ribo1 is a chimeric yeast gene that contains the single intron from the *ACT1* gene and the 3′ end processing signal from *PGK1*. The reporter gene is integrated in the genome, transcribed under the control of a doxycycline-responsive promoter in a doxycycline-inducible strain of *Saccharomyces cerevisiae*. Cells in culture respond to doxycycline in synchrony, and the binding of CTD at five sites (amplicons) along the gene can be measured in this population (see [12] for construct and methods).

**Figure 4.**
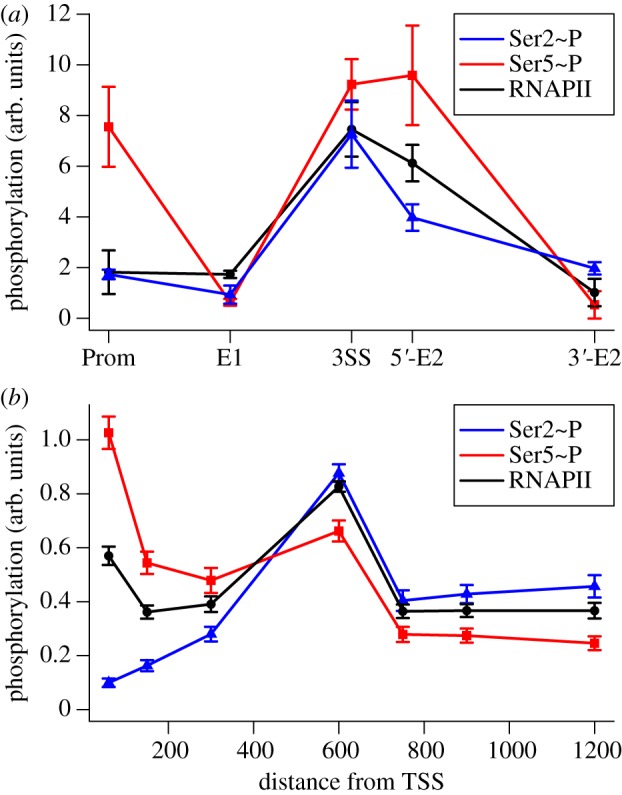
Paused RNAPII, Ser2 and Ser5 phosphorylation in the coding regions of single-intron genes. (*a*) Phosphorylation and RNAPII density along the Ribo1 gene at 240 s after induction (data from [12]). Phosphorylations at the promoter (Prom), exon1 (E1), 3′ splice site (3SS), 5′ end of exon2 (5′-E2) and 3′ end of exon2 (3′-E2) of Ribo1 are indicated. Note that Ser2∼P, Ser5∼P and the density of RNAPII are each normalized to their respective values on induction, and hence are not comparable to each other on an absolute scale. (*b*) Model simulation of Ribo1 with a pause introduced 600 nucleotides downstream of the TSS. (*a*) Real and (*b*) simulated pauses in RNAPII lead to an accumulation of RNAPII that reverses the typical reduction in the absolute level of Ser5∼P and raises the level of Ser2∼P.

RNAPII density, Ser2∼P and Ser5∼P signals along Ribo1 are shown in figure 4*a*, and the results of simulating Ribo1 are shown in figure 4*b*. The model now includes a rule that briefly pauses RNAPII elongation 600 nucleotides from the TSS. The distance of the pause site from the TSS is important as it determines the extent to which Ser2 and Ser5 are phosphorylated, and hence the degree to which phosphorylation will apparently increase on paused RNAPII. This factor is accurately accounted for in the simulation. An increase in the absolute level of phosphorylation is expected where RNAPII pauses; however, additional changes in phosphorylation observed at the 5′ end of exon 2 (see point 5′-E2 in figure 4*a*) that occur concurrently with a decrease in RNAPII are highly significant as they may indicate signalling that occurs subsequent to the pause, rather than changes that can be ascribed to RNAPII density [12].

## 5. Conclusions

Messenger RNA transcription is a complex process with many regulatory interconnections acting through RNAPII subunits. The proposed model of the CTD cycle accounts for both the substrate specificity of the CTD kinases and phosphatases, and the contexts that influence their recruitment. The model was created by surveying and reconciling the evidence in the literature, and this leads to the novel observation that Rtr1 regulates the Ser5∼P to Ser2∼P transition by removing the substrate for the Ser2 kinases on many (but not all) heptads. We demonstrate that the model reproduces the prototypical pattern of phosphorylation observed from the promoter, through to the 3′ UTR, based solely on the binding kinetics of the kinases and phosphatases with the CTD substrate. Modelling suggests that Ctk1 may have a Ser5 kinase activity (as has been demonstrated *in vitro*) as this improves the fit of the model to the data, and provides a mechanism whereby Ser5∼P can be reduced considerably while retaining the possibility of restoring this state as a substrate for the known Ser2 kinases.

Undoubtedly there are other, yet to be discovered, interactions between these proteins and RNAPII, and with each other. The addition of new rules, or the refinement of existing rules, will allow such interactions to be added to the rule-based model in a straightforward fashion. For example, were it to be shown that Bur1 recognizes the bivalent mark Ser5∼P + Ser7∼P as hypothesized in [24], the rules for Bur1 binding could be strengthened to represent this finding.

Rule-based modelling addresses combinatorial expansion in systems modelling, and enables the simulation and analysis of reactions whose formal analysis would otherwise be intractable [2,27,28]. The rule-based approach is being extended to multi-level modelling [29,33], where model entities can be nested, and downward and upward causation between different levels is possible, and to the representation of spatial relations in models [34]. Thus, the approach holds great potential at theoretical and practical levels.

## 6. Material and methods

A detailed Kappa model of 219 rules describing the entire transcription pathway has been constructed (see the electronic supplementary material, file model.tar.gz). This model represents PIC assembly, followed by pre-mRNA capping, elongation, 3′ end maturation and polyadenylation, and release of the mature mRNA [4,6], and is based on a validated stochastic model of a yeast reporter [35]. The complex processing represented in the model yields realistic behaviour in terms of time required for RNAPII to transcribe the simulated gene, and the net synthesis rate of one mature RNA every 4 s previously observed in a yeast reporter [36].

The transcription initiation rules (see the electronic supplementary material, file transcription.ka) define how Activator and Mediator bind to DNA, followed by RNAPII and TFIIH. The phosphorylation of serines 5 and 7 of RNAPII, and capping of the nascent transcript T1 occur during initiation. Rules for elongation specify how RNAPII progresses from one site of DNA to the next, while simultaneously extending the RNA transcript (see the electronic supplementary material, file elongation.ka). Following [35], RNAPII occupies a 30 nt footprint (DNA sites e1–e80 represent spans of 30 nt that RNAPII occupies in sequence) and hence the gene has a maximum capacity for RNAPII. Pausing of RNAPII can cause a queue of polymerase as those behind are prevented from progressing. Rules for the maturation of the RNA through the binding of Pcf11, synthesis of the polyA tail, binding of Rtt103 and release of the mature transcript and RNAPII are defined (see the electronic supplementary material, file maturation.ka). Pcf11 binding requires RNAPII Ser2 to be phosphorylated. Maturation occurs over 20 sites on DNA (pA1–pA20) that RNAPII occupies in sequence until release. The rules for phosphorylation of the CTD are defined in the electronic supplementary material, file phosphorylation.ka. These specify the actions of Bur1, Ctk1, Fcp1, Rtr1 and Ssu72 as described in the text. The binding rates for these proteins are *p0*, *p0/2* or *p0/10* (denoted by phosph0, phosph1 and phosph2, respectively, in the electronic supplementary material, file phosphorylation.ka). The rate for release is *p3* (denoted by phosph3 in the electronic supplementary material, phosphorylation.ka). The binding of Ctk1 to RNAPII sites ser2a and ser2b (representing Ser2 on heptad repeats a and b) is defined in rules binds1 and binds2 below. The combination of RNAPII sites e,a!_ creates a condition that is only met while RNAPII is transcribing the coding region of the gene, namely, e is unbound and a is bound to something (the model allows RNAPII sites a and b to bind to DNA). The subsequent unbinding reactions are release1 and release2. Serine 5 is phosphorylated on release. Two heptapeptide repeats are modelled as this number is sufficient for generating steady-state estimates of phosphorylation from three discrete levels (0, 1 or 2 repeats phosphorylated) obtained from 500 stochastic simulations with good reproducibility and acceptable standard deviation.

'binds1’

Ctk1(a), RNAPII(e,a!_,ser2a^∼^u,ser5a^∼^u) ->

Ctk1(a!1), RNAPII(e,a!_,ser2a^∼^u!1,ser5a^∼^u) @ 'phosph1’

'binds2’

Ctk1(a), RNAPII(e,a!_,ser2b^∼^u,ser5b^∼^u) ->

Ctk1(a!1), RNAPII(e,a!_,ser2b^∼^u!1,ser5b^∼^u) @ 'phosph1’

'release1’

Ctk1(a!1), RNAPII(ser2a^∼^u!1,ser5a^∼^u) ->

Ctk1(a), RNAPII(ser2a^∼^u,ser5a^∼^p) @ 'phosph3’

'release2’

Ctk1(a!1), RNAPII(ser2b^∼^u!1,ser5b^∼^u) ->

Ctk1(a), RNAPII(ser2b^∼^u,ser5b^∼^p) @ 'phosph3’

The CTD kinetics are determined by the structure of the model and a limited number of rate parameters. All agent signatures and rates are defined in the respective files (signatures.ka and rates.ka).

## Endnotes

1 While it is to be expected that the entire dynamic range of phosphorylation that can be predicted (0–100%) may not map to distinct observable values, especially for Ser2∼P which is around 0 at the transcription start site, a better model will maximize this range.
